# The Significance of Epidermal Growth Factor in Noninvasively Obtained Amniotic Fluid Predicting Respiratory Outcomes of Preterm Neonates

**DOI:** 10.3390/ijms23062978

**Published:** 2022-03-10

**Authors:** Violeta Gulbiniene, Greta Balciuniene, Justina Petroniene, Rita Viliene, Irena Dumalakiene, Ingrida Pilypiene, Diana Ramasauskaite

**Affiliations:** 1 Institute of Clinical Medicine, Faculty of Medicine, Vilnius University, M.K. Ciurlionio 21/27, LT-03101 Vilnius, Lithuania; greta.balciuniene@gmail.com (G.B.); ingrida.pilypiene@mf.vu.lt (I.P.); 2 Center of Obstetrics and Gynaecology, Vilnius University Hospital Santaros Klinikos, Santariskiu 2, LT-08661 Vilnius, Lithuania; just.svarauskaite@gmail.com; 3 State Research Institute Centre for Innovative Medicine, Santariskiu 5, LT-08410 Vilnius, Lithuania; ritviliene@gmail.com (R.V.); irena.dumalakiene@gmail.com (I.D.)

**Keywords:** preterm birth, epidermal growth factor, respiratory outcomes, amniotic fluid, noninvasive method, preterm premature rupture of membranes

## Abstract

Preterm premature rupture of membranes (PPROM) interrupts normal lung development, resulting in neonatal respiratory morbidity. Although post-PPROM risks have been researched, only a few studies have investigated noninvasively obtained amniotic fluid (AF) to predict neonatal outcomes. In this study, we aimed to determine whether epidermal growth factor (EGF) in vaginally-collected AF is a significant predictor of neonatal respiratory outcomes after PPROM. We analyzed EGF in vaginally-obtained AF from 145 women with PPROM at 22–34 weeks of gestation. The following neonatal outcomes were included: respiratory distress syndrome, surfactant need, duration and type of respiratory support, and bronchopulmonary dysplasia. We found that EGF concentration was associated with gestational age, and its medians were lower in neonates with respiratory morbidities than unaffected ones. EGF concentrations gradually declined, the lowest being in the most clinically ill patients. EGF < 35 pg/mL significantly predicted the odds of severe respiratory outcomes. EGF in noninvasively collected AF may be a reliable predictor for respiratory outcomes of preterm neonates with PPROM before 34 weeks of gestation. The results of our study may have implications for further research both in noninvasive amniotic fluid analysis and the management of patients after PPROM.

## 1. Introduction

Preterm birth (before 37 weeks of gestation) remains the leading cause of perinatal morbidity and mortality [1]. Most preterm births are spontaneous, with approximately 30% of preterm deliveries following preterm premature rupture of membranes (PPROM) [2]. The assessment and management of women with PPROM have been challenging. The comprehensive treatment strategy depends on the duration of gestation and determining the risk for immediate delivery versus expectant management for both mother and newborn [3].

Morbidity associated with prematurity includes short-term outcomes, such as respiratory distress syndrome (RDS), sepsis, necrotizing enterocolitis, intraventricular hemorrhage, and long-term health consequences, including bronchopulmonary dysplasia (BPD), hearing and visual impairments, neurodevelopmental delay, and cerebral palsy [4,5,6].

Respiratory disorders are among the most frequent causes of morbidity in preterm neonates [7,8,9]. Preterm birth disrupts normal lung development. The respiratory system may be affected by various factors such as intraamniotic infection antenatally and postnatally as sepsis, positive pressure ventilation, and supplemental oxygen [9,10]. An intraamniotic infection has a multifaceted effect on the respiratory system [11]. On the one hand, fetal exposure to bacterial and inflammatory products accelerates lung maturity, thus improving the chances of survival immediately after preterm birth. On the other hand, inflammation increases the risk for long-term outcomes such as BPD due to accompanying pathological changes in the lung anatomy. Nevertheless, even if the premature infant is not experiencing breathing difficulties during the perinatal period, data suggest that respiratory complications may occur later in life [5].

Lung growth and maturation are critical for normal lung formation and, thus, respiration after birth [9]. Tissue interactions and various cell functions essential to lung development are mediated and regulated by growth factors [12].

Epidermal growth factor (EGF) is a small mitogenic polypeptide that belongs to the family of human EGF-related growth factors [13]. EGF is found in most body fluids, including amniotic fluid (AF) [14,15]. The amniotic fluid has high levels of EGF, which is essential to fetal growth and development [16]. EGF acts as a promoter of epithelial cell growth and has an important role in airway branching, stimulating the growth of the epithelial tubules, and in differentiating lung cells during embryonic, fetal, and postnatal lung development [12,17]. Moreover, EGF is involved in lung surfactant synthesis by accelerating the maturation of alveolar type II cells [18,19,20] and producing surfactant protein A [21,22]. Throughout gestation, amniotic fluid EGF levels increase [14]. In animal models, intraamniotic EGF injections decrease the severity and duration of respiratory disease in preterm newborns [22]. Conversely, modified expression of growth factors, such as EGF, has been reported in pathological lung conditions such as bronchopulmonary dysplasia, bronchial asthma, and pulmonary fibrosis [12,13]. Currie et al. indicated decreased levels of EGF in the bronchoalveolar lavage fluid after birth in preterm newborns who developed BPD [13]. 

EGF has been identified as a factor associated with lung growth and respiratory diseases since its discovery in the early 1960s; however, there is a lack of knowledge of whether EGF in amniotic fluid may be a predictive biomarker of respiratory outcomes in preterm neonates. In addition, previous research on EGF has analyzed AF samples obtained only by amniocentesis [14,15,23,24,25,26]. None of the studies analyzing biomarkers in noninvasively collected AF have evaluated EGF concentrations [27,28,29,30,31]. The sensitivity of the noninvasive method and strong correlation between biomarker levels in amniotic fluid collected via amniocentesis or vaginally have been reported by Musilova et al. [30]. In the present study, we aimed to investigate the significance of epidermal growth factor in vaginally obtained amniotic fluid to predict respiratory outcomes in preterm neonates after PPROM.

Since preterm birth and PPROM may be complicated by intraamniotic infection and fetal inflammatory response syndrome [11], we investigated EGF levels associated with histological chorioamnionitis and fetal inflammatory response syndrome. The relationship between EGF and inflammatory cytokines such as interleukin-6 (IL-6), tumor necrosis factor α (TNF-α), and matrix metalloproteinase-8 (MMP-8) were also evaluated. We previously reported the significance of inflammatory biomarkers in noninvasively obtained amniotic fluid predicting histological chorioamnionitis [32]. 

## 2. Results

### 2.1. EGF Median Concentrations in Noninvasively Obtained Amniotic Fluid

**Figure 1:**
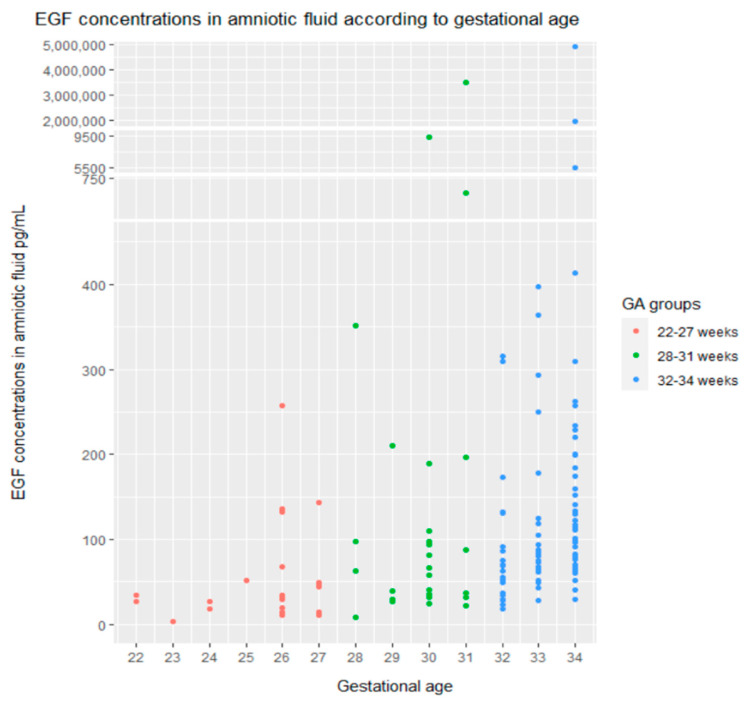
EGF concentrations in the amniotic fluid according to gestational age. *Y*–axis is displayed with breaks to include all values of EGF, even outliers, using the ggbreak R package [33].

EGF was identified in all amniotic fluid samples, with a median of 81.15 pg/mL (interquartile range or IQR: 41.07–133.38). Low EGF concentrations were associated with lower gestational age (GA), and EGF concentrations increased with gestation (Figure 1): 22–27 weeks GA group’s EGF median was 34.20 pg/mL (IQR: 19.67–50.14) vs. 28–31 weeks GA group’s median of 62.62 pg/mL (IQR: 34.95–110.46) vs. 32–34 weeks GA group’s median of 93.36 pg/mL (IQR: 64.89–163.53). The difference between median EGF concentrations in GA groups was statistically significant (*p* < 0.0001). The minimum concentration of 3.56 pg/mL was detected in the AF sample of 23 weeks GA, and the maximum concentration of 489,735 pg/mL was in the AF of 34 weeks GA. The median EGF concentrations among GA groups have risen almost threefold between 22–27 weeks and 32–34 weeks of gestation.

Spearman’s correlation analysis was conducted to assess the association of EGF and other numerical variables. A positive correlation was found between EGF, gestational age, and birth weight. A negative correlation was detected between EGF and the duration of respiratory support (RS), the duration of mechanical ventilation, and the duration of noninvasive respiratory support. However, linear correlations were weak, with Rho coefficients less than 0.5. 

**Figure 2:**
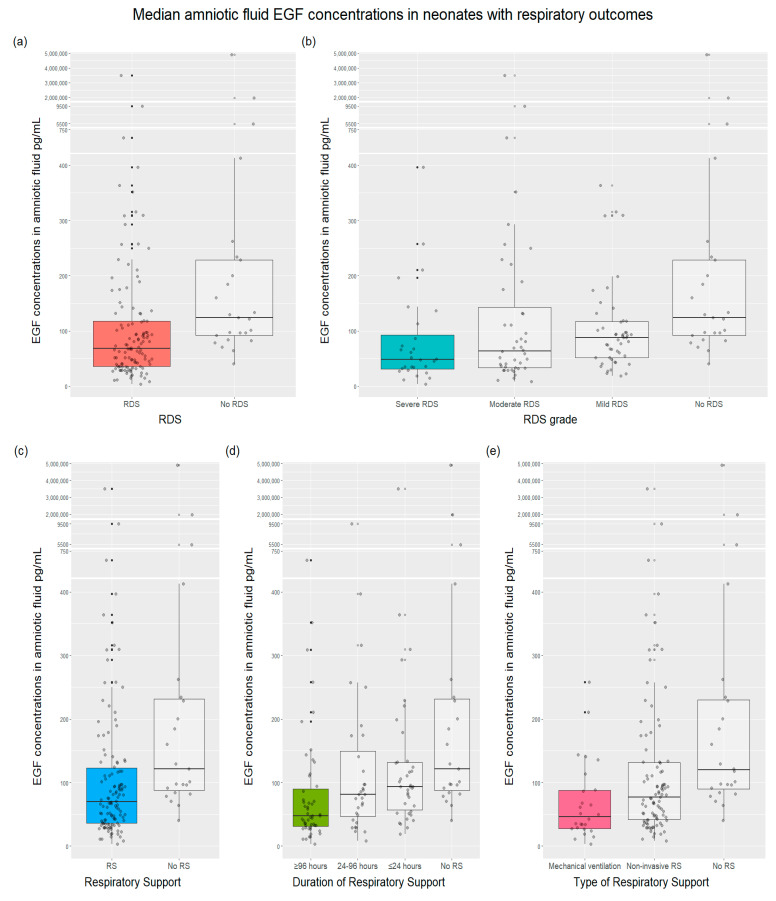

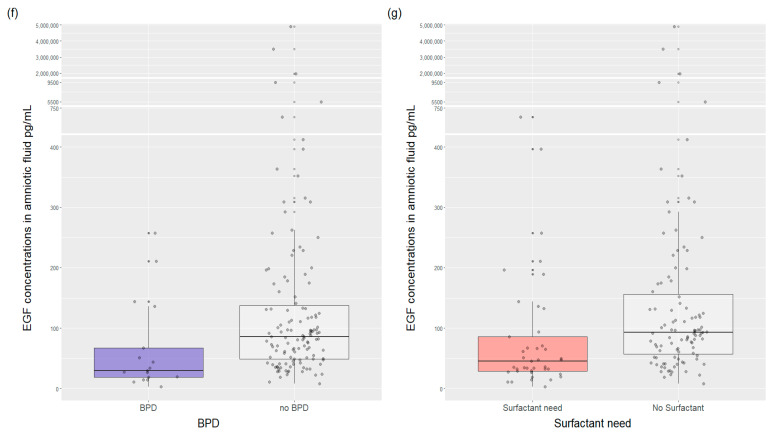
Median amniotic fluid EGF concentrations in neonates with respiratory outcomes compared with neonates without outcomes, (*p* < 0.001 for all assays): (**a**) Median EGF concentrations with RDS vs. no RDS were 68.25 pg/mL vs. 124.29 pg/mL (*p* < 0.001), respectively; (**b**) median EGF concentrations for severe RDS were 48.70 pg/mL vs. 63.80 pg/mL of moderate RDS vs. 88.20 pg/mL of mild RDS vs. 124.00 pg/mL of no RDS (*p* < 0.001); (**c**) median EGF concentrations in infants with a need for respiratory support was 69.85 pg/mL compared with 121.88 pg/mL in infants without RS (*p* < 0.001); (**d**) median EGF concentrations depending on the duration of RS were as follows: 122.00 pg/mL with no RS, 93.50 pg/mL with ≤24 h RS, 81.10 pg/mL with 24–96 h RS, and 47.70 pg/mL with ≥96 h RS (*p* < 0.001); (**e**) median EGF concentrations with a different type of ventilation or no RS were: mechanical ventilation vs. noninvasive RS vs. no RS—46.10 pg/mL vs. 77.10 pg/mL vs. 120.00 pg/mL (*p* < 0.001), respectively; (**f**) median EGF concentrations with BPD vs. no BPD were 29.90 pg/mL vs. 86.06 pg/mL (*p* = 0.0016), respectively; (**g**) median EGF concentrations in newborns with surfactant need vs. no need for surfactant were 45.36 vs. 93.33 pg/mL (*p* < 0.001), respectively. RDS—respiratory distress syndrome; RS—respiratory support; BPD—bronchopulmonary dysplasia.

We performed the analysis to determine whether neonates with respiratory morbidity had different EGF levels in their mother’s amniotic fluid compared with unaffected infants, including RDS severity groups according to chest radiography findings and RS type and duration groups. Median amniotic fluid EGF concentrations were significantly lower in neonates with respiratory outcomes than in infants without it: RDS vs. no RDS, a need for RS vs. no need for RS, BPD vs. no BPD, and the need for surfactant vs. no need for surfactant (Figure 2). We found that EGF levels gradually decreased across severity groups of RDS and RS type and duration groups (Figure 2). 

### 2.2. EGF Predicting Models for Respiratory Outcomes

We assessed the significance of EGF for predicting respiratory outcomes using the univariate logistic regression with different EGF cut-off values. To estimate the odds for respiratory morbidity, we constructed predictive models for severe outcomes, as follows: severe RDS, RS for >4 days, the need for surfactant, mechanical ventilation, BPD. Table 1 presents results obtained from the univariate regression analysis. The logistic regression revealed that low EGF concentrations might predict severe respiratory outcomes. The odds ratio for severe respiratory outcomes increased as the EGF cut-off value decreased. By contrast, the higher the cut-off value of EGF, the lower the odds ratio for severe respiratory morbidity if statistically significant. Overall, in the univariate logistic regression, an amniotic fluid EGF cut-off concentration less than 35 pg/mL significantly predicted and had the highest odds ratios for respiratory outcomes: severe RDS, RS for >4 days, the need for surfactant, mechanical ventilation, and BPD. 

Due to the highest estimated likelihood with an EGF cut-off value of <35 pg/mL in the univariate regression, the multiple regression analysis was performed to investigate a prediction model, adjusting to gestational age (Table 2). The input variable of EGF was not statistically significant in this model. The other input variable of gestational age had a statistically significant impact on the outcomes. For a unit increase in weeks of gestation, the odds for outcomes decreased 0.3–0.7 times, confirming that gestational age is a strong factor in influencing outcomes and determining neonatal risk. 

We noticed that gestational age affects EGF levels and outcomes based on the results presented. To control and investigate GA’s impact on the outcomes, we constructed models with an EGF cut-off value of less than 35 pg/mL (vs. >35 pg/mL) in different GA groups (Table 2). However, estimating the odds of infants with 22–27 weeks GA and 32–34 weeks GA, some models were impossible to run due to zero case events in particular groups: All models with 32–34 weeks GA group, except for mechanical ventilation, 22–27 weeks GA models for the need of surfactant, and RS for >4 days. In possible models of 22–27 weeks GA, outcomes were not significantly predicted by EGF, and GA was a more reliable predictor. The model for mechanical ventilation in 32–34 weeks GA infants with EGF < 35 pg/mL was also not significant. In 28–31 weeks of the GA model, EGF < 35 pg/mL was a reliable predictor of severe respiratory outcomes. The severe respiratory outcomes were about 3–12 times more likely in infants of 28–31 weeks of GA with EGF < 35 pg/mL than in neonates of other GA (22–27 weeks and 32–34 weeks).

Overall, in univariate logistic regression, low EGF concentrations (<35 pg/mL) may predict neonatal respiratory outcomes. After controlling for gestation age, an EGF cut-off value < 35 pg/mL was a reliable predictor of severe respiratory outcomes in 28–31 weeks GA neonates.

### 2.3. EGF Concentrations in Histological Chorioamnionitis, Fetal Inflammatory Response Syndrome

To investigate additional outcomes, such as whether EGF concentrations are affected by intraamniotic infection and inflammation, we analyzed EGF’s relationship to histological chorioamnionitis and fetal inflammatory response syndrome (FIRS). Median concentrations of EGF did not differ significantly in patients with histological chorioamnionitis or without it—80.95 pg/mL vs. 83.66 pg/mL (*p* = 0.699), respectively. The comparison between median EGF concentrations in infants with and without FIRS did not reveal any differences––80.95 pg/mL vs. 84.13 pg/mL (*p* = 0.435), respectively.

Due to the significantly higher rate of histological chorioamnionitis and FIRS in extremely preterm and very preterm neonates, we investigated the association between median EGF levels with intraamniotic infection according to gestational age (Table 3). The analysis results confirmed that EGF concentrations in noninvasively obtained amniotic fluid were not significantly influenced by chorioamnionitis or FIRS in different gestational age groups. This relationship was verified by evaluating Spearman’s correlation between EGF and IL-6, TNF-α, and MMP-8 concentrations in amniotic fluid. There was no statistically significant correlation between EGF and the inflammatory cytokines IL-6, TNF-α, and MMP-8 concentrations.

## 3. Discussion

Our study investigated whether epidermal growth factor in noninvasively obtained amniotic fluid is significant in predicting respiratory outcomes in preterm neonates after PPROM. The possibility of an intraamniotic infection effect on EGF concentrations was also analyzed.

We demonstrated good use of the noninvasive sampling technique for amniotic fluid analysis. To our knowledge, this is the first study to investigate epidermal growth factor in vaginally-collected amniotic fluid. Former research on amniotic fluid EGF obtained samples by amniocentesis [14,15,23,24,25]. Our study strengthens the idea that the noninvasive collecting method may be an alternative for amniocentesis in PPROM.

Previous studies established a close correlation between EGF concentration and gestational age [13,14,25], consistent with our findings. There was a significant change in EGF concentrations in amniotic fluid with increasing fetal age. Low EGF concentrations were associated with lower gestational age. EGF concentrations increased with increasing gestation. The rise of median EGF concentrations among GA groups was almost threefold between 22–27 weeks and 32–34 weeks of gestation. Haigh compared preterm and term gestation, at 30 and 40 weeks, respectively, reporting a 10-fold rise in EGF concentrations [14]. These results support the notion that epidermal growth factor is closely related to gestation. 

Unexpectedly, we did not establish any EGF association with histological chorioamnionitis, FIRS, or inflammatory cytokines such as IL-6, TNF-α, and MMP-8. Shobokshi [34] previously reported elevated EGF concentrations in cases of premature rupture of membranes (PROM) with intraamniotic infection. Firstly, our results may differ due to distinct definitions of intraamniotic infection. In Shobokshi’s study, the intraamniotic infection was defined as positive amniotic fluid culture regardless of histological findings in the placenta. On the other hand, we evaluated histological chorioamnionitis, funisitis, FIRS, and well-known inflammatory markers [11]. Secondly, there is an essential difference in research populations: term pregnancies with PROM vs. PPROM in 22–34 weeks of gestation, respectively. Since EGF is associated with gestation, the results are difficult to compare. Furthermore, Varner’s findings that amniotic fluid EGF levels did not change by chorioamnionitis confirm our results [23].

The association between gestational age and EGF levels provides thought-provoking insights, particularly considering the inverse relationship between gestational age and respiratory morbidities. As previously mentioned, EGF has an essential role in fetal lung development. This factor enhances the growth and differentiation of epithelial lung cells and surfactant synthesis [12,13,17,18,19,20]. Furthermore, EGF significance in neonatal respiratory morbidities, for example, RDS, chronic lung disease, pulmonary hypoplasia, congenital diaphragmatic hernia, has been reported in animal and human research [13,17,22,35,36]. The findings of our study revealed that median amniotic fluid EGF levels were lower in neonates with respiratory morbidities than unaffected neonates. Interestingly, while analyzing RDS severity and RS type and duration groups, we found that EGF concentrations gradually declined across all groups and were the lowest in the most clinically ill patients. Based on these data, the relative deficiency of EGF appears highly associated with increased respiratory morbidity in preterm newborns, indicating its influence on lung development. Altogether, these findings suggest that EGF may be a biochemical indicator of functional postpartum lung maturity for preterm neonates, possibly reflecting respiratory morbidity after birth. This statement corresponds with Goetzman’s conclusion [22] that EGF advances histological and biochemical maturation of lungs in rhesus monkeys, clinically improving respiratory function after preterm birth. The approach is similar to Currie et al.’s [13] study analyzing EGF in bronchoalveolar lavage (BAL) fluid. The most diseased infants who later developed BPD had almost undetectable EGF levels in BAL than controls since the time of their birth.

Although EGF appears to have a considerable impact on normal lung maturation and the development of respiratory diseases of prematurity such as RDS and BPD, little has been done to evaluate EGF as a predictor of neonatal outcomes. Aschner et al. indicated a scarcity of validated biomarkers that predict respiratory disease and are expressed early in the neonatal period, offering the opportunity for effective and targeted interventions to modify outcomes [37]. The results of our study show that low EGF concentrations may be a predictor of severe respiratory outcomes in preterm infants. The lower the cut-off value of EGF, the higher the odds of outcomes. An amniotic fluid EGF cut-off concentration of <35 pg/mL significantly predicted and had the highest odds for respiratory outcomes: severe RDS, respiratory support for more than 4 days, the need for surfactant, mechanical ventilation, and BPD. Currie [13] also indicated that epidermal growth factor in BAL may predispose chronic lung disease in premature infants. However, no data about EGF cut-off values or odds ratio were previously reported. In general, these findings suggest that EGF may predict severe respiratory outcomes in preterm neonates.

EGF is one of the growth factors associated with the pathogenesis of major respiratory outcomes of prematurity—bronchopulmonary dysplasia—through several pathways. Firstly, massive areas of the epithelial barrier are affected in preterm infants with BPD [9,13]; thus, EGF acts directly on the growth and differentiation of epithelial cells in a normal and injured lung. In addition, altered angiogenesis plays a role in the development of BPD [9]. EGF is one of the pro-angiogenic factors secreted by stem cells, such as human amnion epithelial cells, reducing vascular maldevelopment and lung injury in experimental BPD [36]. Moreover, BPD is the disorder of premature lungs, and EGF is highly involved in lung maturation by enhancing surfactant synthesis [19]. Overall, BPD is a complex disease resulting from multiple pathogenetic processes, characterized by various pathological lung components, and influenced by antenatal and postnatal factors [38]. In this study, we found that even antenatal EGF concentrations are related to BPD, suggesting that EGF levels may help indicate the level of lung maturation predicting BPD. Lal et al. [38] agreed upon the need for predictive BPD biomarkers to initiate precautious measures in patients at risk or avoid certain treatment in patients without risk. Most models used to predict BPD are mainly based on clinical symptoms and lack predictive accuracy. Therefore, we think adding the EGF concentration to a predictive model for BPD may be helpful and improve predictive accuracy.

The main strength of our study is our analysis of epidermal growth factor in noninvasively obtained amniotic fluid. In addition, the number of patients in this study is larger than others that examined noninvasive amniotic fluid. Moreover, all mothers and neonates received standardized treatment according to hospital protocols. The attending neonatologist was not aware of the EGF test results; therefore, the management of the neonates was not influenced by the EGF concentration in amniotic fluid. 

We are also conscious of several weaknesses in our study. Firstly, the number of newborns with outcomes corresponding with the number of infants without consequences was uneven in each gestational age group. Thus, we did not have a significant predicting model of sequelae in each gestational age group. Ideally, affected and unaffected newborns would have been evenly matched in all gestational age groups; however, the incidence of respiratory outcomes depends on gestational age and is low in moderate and late preterm infants. Secondly, we evaluated immediate respiratory outcomes after birth until discharge from the hospital. Data regarding EGF’s relationship to adverse long-term respiratory sequelae would improve knowledge about prematurity outcomes. Further research is needed to evaluate the relationship between EGF in amniotic fluid and long-term respiratory outcomes.

In our opinion, our findings provide a better understanding of EGF’s role in respiratory morbidity and may encourage further EGF research, its use in target-specific treatment, and reduce the side effects of nonspecific therapy in neonates through the concept of ‘individualized medicine’. After preterm premature rupture of membranes, there is the possibility to collect and assess amniotic fluid noninvasively and predict neonatal respiratory outcomes even before birth. Thus, it would help stratify neonatal risk earlier and impact the management strategy for both mother and newborn. 

In conclusion, epidermal growth factor in noninvasively collected AF may be a reliable predictor for respiratory outcomes of preterm neonates with PPROM before 34 weeks of gestation. Our findings may have implications for further research in noninvasive amniotic fluid analysis and management of patients with PPROM in general.

## 4. Materials and Methods

### 4.1. Methods

A prospective cohort study was performed in Vilnius University Hospital Santaros Klinikos. A total of 185 pregnant women admitted to the hospital with PPROM at 22–34 weeks of singleton gestation were included. Exclusion criteria were multiple gestations, vaginal bleeding, placenta previa, fetal and neonatal malformations, and non-reassuring fetal status. All patients provided their informed written consent. Our study was approved by the Vilnius Regional Biomedical Research Ethics Committee (No. 158200-17-931-434).

Gestational age was based on the last menstrual period and confirmed or modified by ultrasound scan at 11 + 0 to 13 + 6 weeks of gestation. Premature rupture of membranes (PROM) was diagnosed by speculum vaginal examination verifying amniotic fluid pooling in the back of the vagina or leaking from the cervix. In uncertain cases, PROM was confirmed by the presence of the placental alpha microglobulin-1 protein in the cervicovaginal fluid (AmniSure^®^, QIAGEN, Germantown, MD, USA).

Free leaking amniotic fluid was obtained vaginally with a sterile centrifuge tube every second day. Mucous, bloody samples with less volume were considered invalid and excluded from the study. To reduce contamination and to obtain clear specimens, samples were centrifuged at 3000× *g* rpm for 5 min at 4 °C and stored at −80 °C. Immunological assays of amniotic fluid samples were performed by the enzyme-linked immunosorbent assay (ELISA) with commercial Human EGF ELISA kits (Bender MedSystems, Vienna, Austria). For the ELISA assay, 1:5 diluted samples were used to determine EGF concentrations. If the measured concentrations of analytes exceeded the highest point on the standard curve, dilutions 1:10 or 1:100 were performed. According to the kit’s inserts, the analytical sensitivity of human EGF was <1 pg/mL. Immunological AF assays of IL–6, TNF-α, and MMP–8 were performed by ELISA, as previously described [32]. 

The newborn’s evaluation after birth and the follow-up assessment before discharge from the hospital was conducted. The newborn’s medical records were also reviewed. The following outcomes were included: respiratory distress syndrome (RDS), surfactant need, the duration (in days) and type of respiratory support (mechanical ventilation, noninvasive respiratory support, and no respiratory support), bronchopulmonary dysplasia, and fetal inflammatory response syndrome (FIRS). 

According to national RDS management guidelines adopted from European guidelines [39], RDS was diagnosed using a combination of respiratory symptoms in a preterm newborn needing respiratory support after delivery, and findings on chest radiography, including low lung volumes with a ‘reticulogranular’ appearance and air bronchograms [40]. The severity of RDS was determined according to chest radiography findings: no RDS—no typical findings, mild RDS (Grade 1)—a diffuse, linear granular pattern; moderate RDS (Grade 2)—bilateral widespread more prominent air bronchograms, severe RDS (Grade 3 and 4)—the opaque lungs and/or alveolar shadowing obscuring the cardiac border [40].

According to European guidelines, surfactant therapy is provided to all neonates ≤ 26 weeks GA prophylactically and for infants > 26 weeks GA based on clinical evaluations of breathing and an inspired oxygen requirement of >0.3–0.35 (very early in the clinical course) [39]. Bronchopulmonary dysplasia was diagnosed based on the need for oxygen supplementation or respiratory support at 28 days postnatal age, 36 weeks postmenstrual age, or until discharge to home. FIRS was defined according to the umbilical cord blood interleukin-6 level > 11 ng/mL and/or histological funisitis. All postpartum placentas were examined histologically. All pregnancies were managed expectantly with full prenatal care, antibiotics, and a single course of antenatal corticosteroids. Participation in the study did not modify our routine clinical care.

### 4.2. Study Population

A total of 145 women and their neonates were enrolled. We eliminated 40 patients due to an invalid or insufficient volume of amniotic fluid samples for ELISA assay; several cases were excluded due to major congenital anomalies diagnosed later in the neonatal period. 

The study population was divided into three gestational age groups according to the WHO classification [41]: extremely preterm infants (22–27 weeks GA), very preterm (28–31 weeks GA), and moderate and late preterm infants (32–34 weeks GA). The demographic and clinical characteristics of the total cohort and subgroups are shown in Table 4 and Table 5. Maternal age was slightly different among the groups. Gravidity, parity, latency period, comorbidities (hypertensive disorders, gestational anemia), and a positive group B streptococcus test did not differ significantly in the subgroups. Gestational diabetes was diagnosed more often in the moderate and late preterm groups. Histological chorioamnionitis and funisitis were common in patients with smaller GA. Neonatal birth weight, Apgar scores less than seven after the 1st and 5th min, FIRS, early-onset sepsis, and late-onset sepsis were statistically different in GA groups. The smaller the GA, the more risk factors and outcomes were present. Umbilical cord arterial pH did not differ significantly.

Table 5 shows neonatal respiratory outcomes in three groups of gestational ages 22–27 weeks, 28–31 weeks, and 32–34 weeks. As expected, the incidence rate of respiratory disorders was different among the groups. Lower gestational age was strongly associated with a higher rate of respiratory outcomes, with a *p*-value of <0.05. The incidence of all adverse neonatal respiratory outcomes as BPD, the severity of RDS, the need for surfactant, and the duration of respiratory support decreased with increasing gestational age. 

### 4.3. Statistics

The data were analyzed using R software version R–4.0.5. (R Core Team, 2020) [42]. The analyses were performed both on the whole population and as subgroup analyses. The Shapiro–Wilk test determined the distribution of the data. General population data were compared between the three subgroups using Student’s t, Mann–Whitney–Wilcoxon, Kruskal–Wallis, χ^2^, or Fisher’s exact tests as appropriate. Parametric continuous variables are expressed as means with standard deviations. Nonparametric variables are provided as a median and interquartile range (IQR), and categorical variables are presented as frequencies and percentages. Correlations were analyzed with Spearman’s coefficient (ρ). Box plots involving the median EGF concentrations (dark horizontal line) with the interquartile range and a scatterplot of raw data distribution were produced. The univariate and multiple regression analysis was used to evaluate the reliability of EGF to predict respiratory outcomes; coefficients, standard errors, and odds ratio were reported with confidence intervals (CI). A *p*-value of <0.05 was considered statistically significant.

## Figures and Tables

**Table 1:** The regression analysis for severe respiratory outcomes with various cut-off values of EGF: respiratory outcomes as the outcome variable, different cut-off values of EGF as the input variable. Significant results are bolded. OR—odds ratio, CI—95% Confidence Interval; RDS—respiratory distress syndrome; RS—respiratory support; BPD—bronchopulmonary dysplasia.

Cut-Off EGF (pg/mL)	Respiratory Outcomes	Estimate	Standard Error	*p*-Value	OR	CI
<200	Severe RDS	0.6004	0.6568	0.36	1.82	0.57–8.15
	The need for Surfactant	0.8015	0.5823	0.17	2.23	0.78–8.06
	RS for >4 days	0.8596	0.5365	0.11	2.36	0.89–7.51
	Mechanical ventilation	1.0012	0.7729	0.20	2.72	0.73–17.72
	BPD	0.4520	0.7884	0.57	1.57	0.40–10.42
<100	Severe RDS	0.6286	0.4760	0.19	1.88	0.77–5.08
	The need for Surfactant	0.9354	0.4269	**0.03**	2.55	1.14–6.17
	RS for >4 days	0.8972	0.3910	**0.02**	2.45	1.14–6.17
	Mechanical ventilation	0.7422	0.5021	0.14	2.10	0.83–6.09
	BPD	0.6804	0.6003	0.26	1.97	0.66–7.32
<90	Severe RDS	1.0815	0.4740	**0.02**	2.95	1.21–7.97
	The need for Surfactant	1.2675	0.4139	**<0.01**	3.55	1.62–8.33
	RS for >4 days	1.3103	0.3832	**<0.001**	3.71	1.78–8.08
	Mechanical ventilation	1.1872	0.5002	**0.02**	3.28	1.29–9.49
	BPD	1.0999	0.5987	0.07	3.00	1.001–11.12
<75	Severe RDS	1.3146	0.4589	**<0.01**	3.72	1.56–9.64
	The need for Surfactant	1.6245	0.4086	**<0.0001**	5.08	2.34–11.71
	RS for >4 days	1.7634	0.385	**<0.0001**	5.44	2.79–12.75
	Mechanical ventilation	1.1676	0.4639	**0.02**	3.21	1.33–8.38
	BPD	1.5124	0.5994	**0.01**	4.54	1.51–16.81
<50	Severe RDS	1.1635	0.4330	**<0.01**	3.20	1.37–7.59
	The need for Surfactant	1.6482	0.3978	**<0.0001**	5.20	2.41–11.54
	RS for >4 days	1.6942	0.3888	**<0.0001**	5.44	2.58–11.89
	Mechanical ventilation	1.1543	0.4444	**0.01**	3.17	1.33–7.70
	BPD	1.6124	0.5457	**<0.01**	5.01	1.77–15.56
<35	Severe RDS	1.1170	0.4703	**0.02**	3.06	1.19–7.65
	The need for Surfactant	1.9849	0.4580	**<0.0001**	7.28	3.03–18.47
	RS for >4 days	1.6275	0.4570	**<0.001**	5.09	2.13–12.97
	Mechanical ventilation	1.4816	0.4756	**<0.01**	4.40	1.72–11.24
	BPD	2.2239	0.5575	**<0.0001**	9.24	3.15–28.76

**Table 2:** The multiple regression analysis for respiratory outcomes with EGF cut–off level < 35 pg/mL, adjusted for gestational age. Significant results are bolded. aOR—adjusted odds ratio, CI—95% Confidence Interval; GA—gestational age; RDS—respiratory distress syndrome; RS—respiratory support; BPD—bronchopulmonary dysplasia.

Outcome Variable	Input Variable	Coefficients	Estimate	Std. Error	*p*-Value	aOR	CI
Severe RDS	EGF < 35 pg/mL + GA	EGF < 35	−0.3572	0.6234	0.567	0.7	0.19–2.26
		GA	−0.4072	0.0874	**<0.0001**	0.7	0.56–0.78
The need for Surfactant		EGF < 35	0.5996	0.6356	0.345	1.82	0.51–6.28
		GA	−0.6879	0.1179	**<0.0001**	0.5	0.39–0.62
RS for >4 days		EGF < 35	−0.0796	0.6755	0.906	0.92	0.23–3.38
		GA	−0.8263	0.1411	**<0.0001**	0.44	0.32–0.56
Mechanical ventilation		EGF < 35	0.2768	0.5926	0.641	1.32	0.39–4.09
		GA	–0.3524	0.08561	**<0.0001**	0.7	0.59–0.83
BPD		EGF < 35	0.3220	0.9240	0.73	1.38	0.19–2.26
		GA	–1.286	0.363	**<0.0001**	0.28	0.11–0.47
Severe RDS	EGF < 35 pg/mL + 22–27 weeks GA **	EGF < 35	0.2361	0.5796	0.68	1.27	0.38–3.77
		GA	2.2752	0.5507	**<0.0001**	9.73	3.38–30.00
Mechanical ventilation		EGF < 35	0.6753	0.5704	0.24	1.96	0.61–5.84
		GA	2.2915	0.5511	**<0.0001**	9.9	3.41–30.19
BPD		EGF < 35	1.1423	0.8741	0.191	3.13	0.57–19.15
		GA	5.3143	1.1132	**<0.0001**	203.21	33.3–3997.7
Severe RDS	EGF < 35 pg/mL + 28–31 weeks GA	EGF < 35	1.0703	0.4744	**0.024**	2.92	1.13–7.35
		GA	0.5004	0.4962	0.31	1.65	0.60–4.26
The need for Surfactant		EGF < 35	1.9495	0.4616	**<0.0001**	7.03	2.90–17.94
		GA	0.6295	0.4737	0.184	1.88	0.73–4.72
RS for >4 days		EGF < 35	1.5894	0.4730	**<0.001**	4.9	1.98–12.86
		GA	1.3609	0.4551	0.003	3.9	1.62–9.76
Mechanical ventilation		EGF < 35	1.6155	0.4928	**0.001**	5.03	1.91–13.41
		GA	–1.0486	0.6885	0.13	0.35	0.07–1.19
BPD		EGF < 35	2.5074	0.5912	**<0.0001**	12.27	3.95–41.13
		GA	–2.1194	1.1082	0.056	0.12	0.006–0.72
Mechanical ventilation	EGF < 35 pg/mL + 32–34 weeks GA **	EGF < 35	1.0043	0.5185	0.052	2.73	0.98–7.54
		GA	–1.1414	0.4956	**0.02**	0.32	0.12–0.84

** The regression analysis was not performed due to zero events in particular groups, as follows: the need for surfactant, RS for >4 days—in 22–27 weeks GA; severe RDS, the need for surfactant, RS for >4 days, BPD—in 32–34 weeks GA.

**Table 3:** Fetal inflammatory response syndrome (FIRS) and histological chorioamnionitis (HCA) effects on median EGF concentrations (pg/mL) in gestational age groups.

	22–27 Weeks		28–31 Weeks		32–34 Weeks	
	Median EGF	*n*	Median EGF	*n*	Median EGF	*n*
FIRS	32.7	11	41.1	15	93.4	65
No FIRS	34.4	13	84.6	14	91.6	27
*p*-value	0.931		0.513		0.764	
HCA	32.7	9	38.1	18	92.5	64
No HCA	34.4	15	97.1	11	103.	28
*p*-value	0.976		0.065		0.715	

**Table 4:** Demographic and clinical characteristics of the total study population, and three gestational age groups, separately. The data are presented as median (interquartile range) or mean with standard deviation for continuous variables and as number (percent) for categorical variables. Significant *p*-values are written in bold. GBS—a group B streptococcus test; GA—gestational age; FIRS—fetal inflammatory response syndrome; EOS—early-onset sepsis; LOS—late-onset sepsis.

	Total Cohort (*n* = 145)	22–27 Weeks (*n* = 24)	28–31 Weeks (*n* = 29)	32–34 Weeks (*n* = 92)	*p*-Value
Mothers’ characteristics					
Age of mother (years)	31.25 ± 5.62	29.8 ± 4.1	32.9 (±5.74)	31.1 (±5.82)	**0.048**
Latency period (hours)	17.7 (6.2–51)	28.33 (12.2–60.3)	15.87 (4–40.6)	17.44 (6.03–52.2)	0.321
Hypertensive disorders	31 (21.38%)	4 (16.7%)	9 (31%)	18 (19.6%)	0.358
Gestational Diabetes	34 (23.61%)	2 (8.3%)	4 (14.3%)	28 (30.4%)	**0.035**
Gestational anemia	38 (26.21%)	4 (16.7%)	9 (31%)	25 (27.2%)	0.502
GBS positive	17 (11.72%)	2 (8.3%)	4 (13.8%)	11 (12.0%)	0.721
Gravidity:					
Primigravida	50 (34.48%)	8 (33.3%)	10 (34.5%)	32 (34.8%)	0.991
Multigravida	95 (65.52%)	16 (66.7%)	19 (65.5%)	60 (65.2%)	
Parity:					
Primiparous	68 (46.9%)	9 (37.5%)	14 (48.3%)	45 (48.9%)	0.599
Multiparous	77 (53.1%)	15 (62.5)	15 (51.7%)	47 (51.1%)	
Histological chorioamnionitis	54 (37.24%)	15 (62.5%)	11 (37.9%)	28 (30.4%)	**0.015**
Funisitis	21 (14.48%)	5 (20.8%)	8 (27.6%)	8 (8.7%)	**0.004**
Neonates’ characteristics					
GA at birth (weeks)	32 (30–34)	26 (25.8–27)	30 (29–30)	33 (33–34)	**<0.0001**
Birth weight (grams)	1840 (±634)	868(±205)	1438 (±264)	2221 (±396)	**<0.0001**
Apgar scores < 7 at 1 min.	22 (15.17%)	14 (58.3%)	5 (17.2%)	3 (3.3%)	**<0.0001**
Apgar scores < 7 at 5 min.	7 (25%)	6 (85.7%)	1 (3.4%)	0	**<0.0001**
Umbilical cord arterial pH	7.34 (7.28–7.39)	7.38 (7.25–7.42)	7.32 (7.26–7.4)	7.34 (7.29–7.39)	0.462
FIRS	54 (37.24%)	13 (54.2%)	14 (48.3%)	27 (29.3)	**0.032**
Neonatal death	1 (0.69%)	1(4.2%)	0	0	
EOS	9 (6.2%)	6 (25%)	2 (6.9%)	1(1.1%)	**<0.0001**
LOS	6 (4.13%)	4 (17.4%)	1(3.4%)	1(1.1%)	**0.005**

**Table 5:** The analysis of respiratory outcomes according to gestational age. RS—respiratory support; RDS—respiratory distress syndrome; BPD—bronchopulmonary dysplasia.

Respiratory Outcomes	Total Cohort *n* = 145	22–27 Weeks *n* = 24	28–31 Weeks *n* = 29	32–34 Weeks *n* = 92	*p*-Value
Respiratory distress	121 (83.45%)	24 (100%)	29 (100%)	68 (73.9%)	**<0.0001**
The need for Surfactant	41 (28.47%)	23 (95.8%)	12 (41.4%)	6 (6.6%)	**<0.0001**
Duration of RS:					
no RS	23 (15.86%)	0	0	23 (25.0%)	**<0.001 ^2^**
≤24 h	38 (26.39%)	0	1 (3.4%)	37 (40.2%)	
24–96 h	32 (22.22%)	0	10 (34.5%)	22 (23.9%)	
≥96 h	51 (35.42%)	23 (100%) ^1^	18 (62.1%)	10 (10.9%)	
Method of RS:					
Mechanical ventilation	26 (17.93%)	14 (58.3%)	3 (10.3%)	9 (9.8%)	**<0.0001 ^2^**
Non–invasive RS	95 (65.52%)	10 (41.7%)	26 (89.7%)	59 (64.1%)	
no RS	24 (16.55%)	0	0	24 (26.1%)	
RDS grade:					
No RDS	25 (17.24%)	0	0	25 (27.2%)	**<0.001 ^2^**
Mild RDS	48 (33.1%)	2 (8.3%)	7 (24.1%)	39 (42.4%)	
Moderate RDS	44 (30.34%)	8 (33.3%)	14 (48.3%)	22 (23.9%)	
Severe RDS	28 (19.31%)	14 (58.3%)	8 (27.6%)	6 (6.5%)	
BPD	17 (11.81%)	16 (69.6%)	1 (3.4%)	0	**<0.0001**

^1^ One neonatal death on the first day of life; thus, a total duration of RS is unknown: *n* = 23 of 24 (100%). ^2^ Simulated *p*-value by Monte Carlo simulation [42].

## Data Availability

The data supporting our reported results are available upon request.
